# The emerging role of geropathology in preclinical aging studies

**DOI:** 10.1080/20010001.2017.1304005

**Published:** 2017-03-21

**Authors:** Warren Ladiges

**Affiliations:** ^a^ Department of Comparative Medicine, University of Washington, Seattle, WA, USA

In 2015, the United States National Institute on Aging funded the Geropathology Initiative, designed to develop a systematic approach for using the pathology of aging as a way to assess anti-aging interventions. As a result, the Geropathology Research Network was formed, consisting of various working groups composed of experts in anatomic pathology, molecular pathology, and translational geroscience. Within the Anatomic Pathology Working Group, a Geropathology Grading Committee was formed for the purpose of developing guidelines for a scoring system based on the increasing severity of lesions associated with increasing age, using the mouse as the preclinical prototype model.

The committee consists of a chair, Warren Ladiges, DVM, MSc, a program coordinator, John Morton, BS, from the Department of Comparative Medicine, University of Washington, Seattle, WA, and six board-certified veterinary pathologists: Denny Liggitt, DVM, PhD and Jessica Snyder, DVM, PhD, from the Department of Comparative Medicine, University of Washington, Seattle, WA, Tim Snider, DVM, PhD, from the Department of Veterinary Pathology, Oklahoma State University, Stillwater, OK, Erby Wilkinson, DVM, PhD, from the Department of Pathology, University of Michigan, Ann Arbor, MI, Denise Imai, DVM, PhD, from the Department of Comparative Pathology, University of California, Davis, Davis, CA, and Smitha Pillai, DVM, PhD, from the Fred Hutchinson Cancer Research Center, Seattle, WA.

This committee has been actively engaged in teleconferences, meetings, and workshops to develop the Geropathology Grading Platform (GGP). The GGP is based on a standard set of guidelines designed to (1) detect the histological presence or absence of low-impact lesions; and (2) determine the level of severity of high-impact lesions in organs from aged mice [1]. The platform is designed to generate a numerical score for each lesion in a specific organ, so that a total lesion score is obtained by adding each lesion score for that organ for one mouse. Total lesion scores are averaged between all mice in a specific cohort to obtain a composite lesion score (CLS) for that organ. The CLS can then be used to compare responses to drug treatment over time, determine the effect of alterations in gene expression, or investigate the impact of environmental challenges in a variety of preclinical aging studies [2]. This platform has been used to compare CLS in two different mouse strains at increasing ages, showing that CLSs increase similarly in both strains with increasing age but at different rates in different organs [3]. The platform also showed that middle-aged mice treated with the anti-aging drug rapamycin for 2 months had lower CLSs than mice treated with placebo. CLSs correlate well with other measures of aging, such as chronic progressive heart disease defined by an increasing left ventricular mass index [3], and an age-dependent increase in carpal joint lesions in association with a decrease in grip strength of the front paw [4]. These observations help to establish the value of the GGP as a measure of biological aging aligned with mouse lifespan studies and physiological findings [5,6].

**Figure 1.**
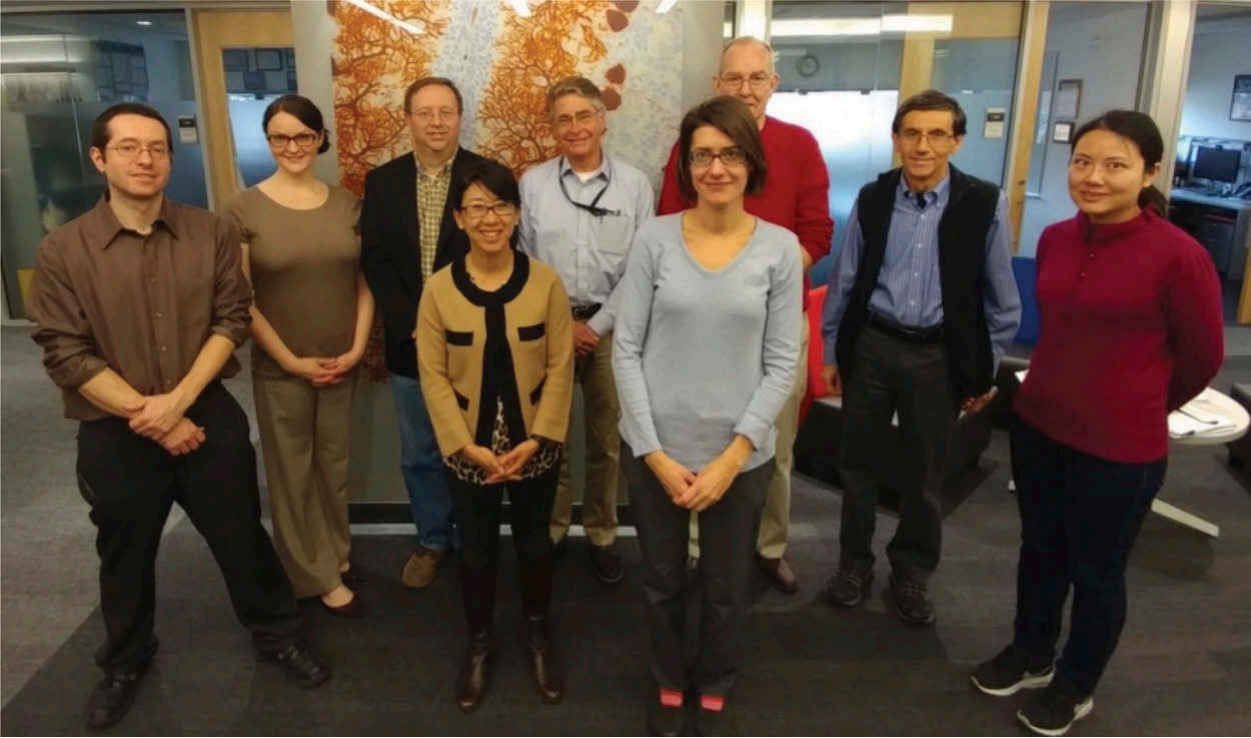
2017 Geropathology Workshop participants. From left to right: John Morton, Dr. Sarah Rostad, Dr. Tim Snider, Dr. Denise Imai, Dr. Denny Liggitt, Dr Jessica Snyder, Dr. Erby Wilkinson, Dr. Warren Ladiges, and Dr. Gigi Ge. Dr. Smitha Pillai attended the workshop but was not present at the time of the photograph.

**Figure 2.**
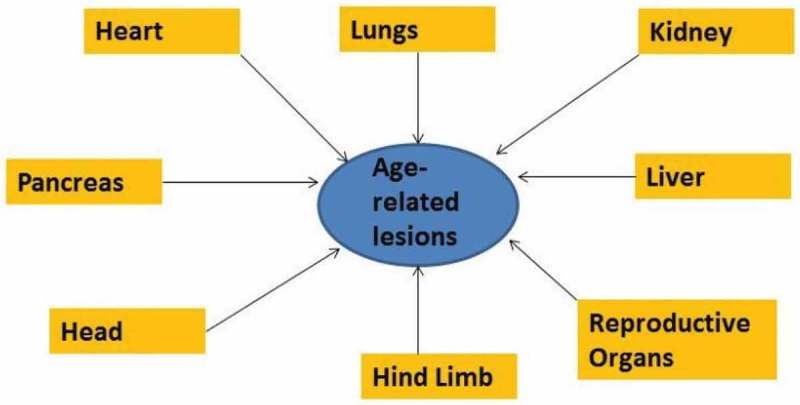
The Geropathology Grading Platform uses various organs and tissues to generate composite lesion scores. These now include the heart; lungs; kidney; liver; head, consisting of brain, nasal cavity, eyelid, teeth, Harderian glands, and inner ear; hind limb, consisting of joint, skeletal muscle, bone, and bone marrow; reproductive organs; and pancreas.

The organs used in these initial assessments were heart, lungs, paw, liver, and kidney, and were shown to have highly representative age-associated lesions. Additional organs and tissues would help to increase the scoring leverage. In this regard, the Geropathology Grading Committee participated in a workshop in January 2017. This was a hands-on workshop in the Department of Comparative Medicine’s eight-headed microscope room, on the University of Washington campus in Seattle, WA, enabling the reading of slides by a small group of pathologists and trainees (Figure 1). The two trainees attending the workshop were Sarah Rostad, DVM, PhD, from the Department of Veterinary Pathology, Oklahoma State University and Oklahoma Medical Foundation, Oklahoma City, and Gigi Ge, MD, PhD, from the Department of Comparative Medicine, University of Washington. A large viewing screen showing the slide being examined was available in the room so that additional individuals could be accommodated. Over a 2 day period a large number of slides were read and a consensus was reached on grading scores and descriptive guidelines for (1) the head, consisting of brain, nasal cavity, eyelid, teeth, Harderian glands, and inner ear; (2) the hind limb, consisting of joint, skeletal muscle, bone, and bone marrow; (3) the reproductive organs; and (4) the pancreas. These are currently being added to the GGP so that a CLS can be generated for them as well (Figure 2).

**Figure 3.**
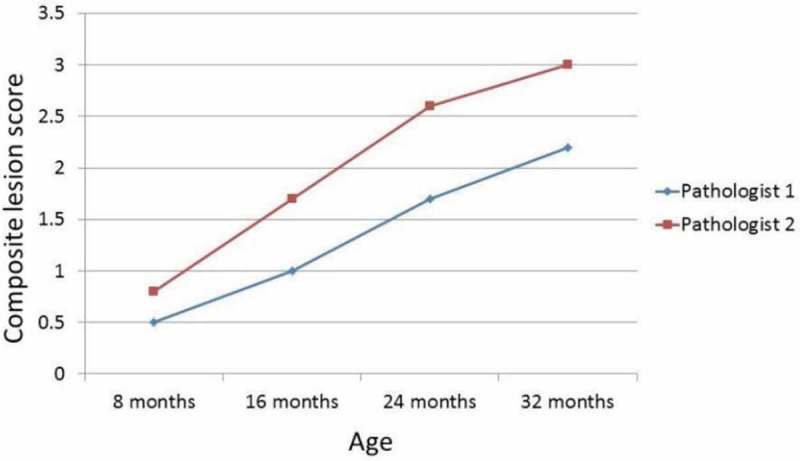
Composite lesion scores of kidneys from four age groups of C57BL/6Jnia male mice generated by two different pathologists increase in a similar manner with increasing age (*n* = 12/age group).

**Figure 4.**
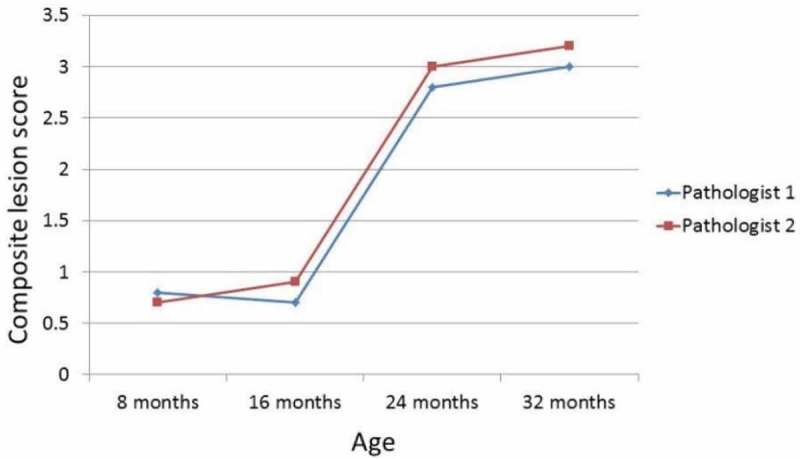
Composite lesion scores of liver from four age groups of C57BL/6Jnia male mice generated by two different pathologists increase in a similar manner with increasing age (*n* = 12/age group).

A second objective of the workshop was to establish the duplicatability of the GGP, i.e. to see whether the generation of CLSs by different pathologists could be duplicated. Figures 3 and 4 show that CLSs were consistent among three different pathologists who blindly read the same liver or kidney slides from four age groups of C57BL/6Jnia mice. These preliminary observations provide evidence that the platform can be duplicated, but more comparisons are needed to confirm these initial findings.

Since the GGP is a new histological grading system for assessing the presence and severity of lesions in tissues from aged mice, few pathologists are familiar with the system. Therefore, a third objective of the workshop was to develop a plan to expand the Geropathology Research Network website (http://www.geropathology.org/) to provide teaching tools as well as the potential infrastructure for integrating the pathology of aging in mice. Several training tools have been posted, including ‘Tissue collection guidelines’ and ‘Necropsy protocol’. Various geropathology lesions are also featured on a regular basis. Plans were discussed to make the website more interactive. The committee also discussed an outline for a Mouse Geropathology Atlas and set future dates for working on this.

In summary, the Geropathology Grading Platform provides a way to measure biological aging in mice, i.e. how quickly a particular organ or tissue ages, which will be applicable to other animal models as well. As such, it is evolving into a critical tool in preclinical intervention studies to determine whether a particular drug or combination slows aging. This is possible since intervention can be started in middle-aged mice and continued for several months to see whether the intervention kept the mice at a younger biological age, as determined by a comparison of lesion scores at the end of the short-term treatment. The Geropathology Grading Committee is a highly motivated group committed to the further development of the GGP as a useful and productive paradigm for helping to increase the efficiency and translational relevance of preclinical aging intervention studies.
